# A case of a Jordanian male twin with Cohen's syndrome, with genetic analysis and muscle biopsy; case report

**DOI:** 10.1016/j.amsu.2021.103014

**Published:** 2021-11-03

**Authors:** Ansam Ghzawi, Hawazen Hirbawi, Ahmad Negida, Hussam Abu-Farsakh

**Affiliations:** a Faculty of Medicine, Yarmouk University, Irbid, Jordan; b Faculty of Medicine, Jordan University of Science and Technology, Irbid, Jordan; c School of Pharmacy and Biomedical Sciences, University of Portsmouth, Portsmouth, UK; d First Medical Lab of Histopathology, Amman, Jordan

**Keywords:** Cohen syndrome, Muscle biopsy, Genetic analysis, Male twins. case report

## Abstract

**Introduction and importance:**

Cohen's syndrome is a rare autosomal recessive developmental disorder. It usually presents with a wide variety of muscular, neurological and ophthalmological clinical features. In this report, we present a rare case of the first Jordanian male identical twin with Cohen syndrome with the first ever muscle biopsy results reported.

**Case presentation:**

A 20 months old identical male twins were presented for follow up with history of Salam seaziure, muscle dystocia and signs of development delay since five months old. A muscle biopsy and genetic analysis were done accordingly. Under light microscopy, the H&E and Trichome stains sections showed muscle fibers with minimal variation in muscle fiber size. No muscular degeneration, fat replacement, or fibrosis in the periendomysial area. Increased fibroblasts proliferation was seen in between the muscle fibers. The Dystrophy panel including Dystrophin, Dysferlin, Adhalin (alpha 1 sacroglycan) and Emerin showed a normal staining pattern. The heterozygous mutation pattern seen in the vacuolar protein sorting 13 homolog B (VPS13B) gene is a pathogenic variant of Cohen syndrome. The diagnosis was done accordingly.

**Conclusion:**

To the best of our knowledge, this is the first case report of Cohen's syndrome from the Jordanian population, and the first muscle biopsy report in a Cohen's syndrome patient ever. This makes a unique educational report and a good guidance for future research in this concern.

## Highlights

• Cohen's syndrome is a rare autosomal recessive developmental disorder.
• Cohen's syndrome is a rare genetic disorder that presents with developmental delay, intellectual disability, and eye manifestations.
• The usual method of testing or screening for Cohen syndrome is VPS13B next-generation sequencing.

## 1 Introduction

Cohen syndrome is a rare autosomal recessive developmental disorder. It was first described by Cohen and colleagues in 1976. [1] It presents with a variety of clinical features including hypotonia, abnormal fat distribution, developmental delay, intellectual disability, coarse facial features, and limb deformities. [2] This is caused by an aberration in the 13B vacuolar sorting protein gene, VPS13B (8q22-8q23). This muted gene plays a role in intracellular protein trafficking and vesicle-mediated sorting. [3] The usual method of testing or screening for Cohen syndrome is VPS13B next-generation sequencing. In this report we present a very rare case of a Jordanian identical twin with Cohen syndrome, with a very unique muscular biopsy histopathological results and a discussion on the available literature. This case has been reported according to SCARE criteria for case reports [4].

## 2 Case presentation

This study describes the clinical and microscopic findings in male identical twin patients who are one year and eight months of age. The twins experienced a normal pregnancy and were born to first-degree consanguineous parents. They were doing well until five months after delivery when they started to have salaam seizures along with muscle dystonia. They also showed decreased mental development when compared to children their age. They do not talk or walk and are unable to say any clear word. The twins do not recognize their parents and still cannot sit properly. The twins are eating well and have no history of dysphagia, urinary tract infection, or chest infections. There is no family history of the same disease. Their elder sister is normal. On clinical examination, the twins displayed hypotonia in their neck and limbs along with increased tendon reflexes.

**Fig. 1:**
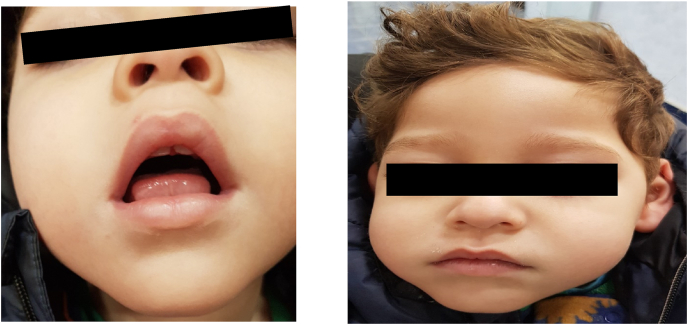
Downward-slanting and wave-shaped palpebral fissures, thick eyebrows and eyelashes, a prominent, beak-shaped nose with a high nasal bridge, malar hypoplasia Maxillary prognathia, with open mouth and protruding tongue.

**Fig. 2:**
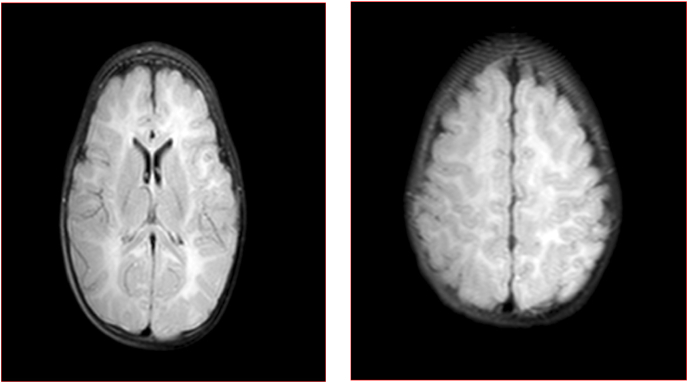
(A, B): MRI shows high signal intensity in periventricular area, up to the cortex with mild brain atrophy.

They had thick hair and a low hairline, downward-slanting and wave-shaped palpebral fissures, thick eyebrows and eyelashes. They also had a prominent beak-shaped nose with a high nasal bridge, malar hypoplasia and a high-arched palate. The philtrum is short and upturned with maxillary prognathia, an open mouth expression due to labial incompetence (Fig. 1). Further tests show the episcleral venous pressure was found to be slightly decreased. The EEG showed salaam seizure features, which the twins still experience every day. CPK levels were normal. MRI showed a diffuse increased white matter density involving the periventricular areas and extending to the subcortical white matter (Fig. 2A and B).

## 3 Microscopic findings

Multiple needle biopsy pieces from the right quadriceps muscle were obtained by a special muscle biopsy needle. Some of the pieces were put in formalin for light microscopy (H&E and modified Trichome stain) and the others were put in a saline-wet gauze and then submitted for −70° freezing with liquid nitrogen. Examination and staining were performed using the following stains: H&E, ATPase at PH: 9.4; PH: 4.6, and PH: 4.2. NADH, succinate dehydrogenase, and modified Trichome stain.

Under light microscopy, the H&E and Trichome stains sections showed muscle fibers with minimal variation in muscle fiber size. No muscular degeneration, fat replacement, or fibrosis in the periendomysial area. No vacuolation of the muscle fibers was noted. Increased fibroblasts proliferation was seen in between the muscle fibers (Fig. 3A). No phagocytosis of the muscle fibers, muscle necrosis, or hyaline fibers was noted. No split fibers, muscle inflammation, group atrophy of nuclei, or perifascicular atrophy were noted. No angulated atrophic fibers were seen. Internalization of nuclei was noted in less than 5% of the fibers. No inclusions were noted by the Trichome stain.

**Fig. 3A:**
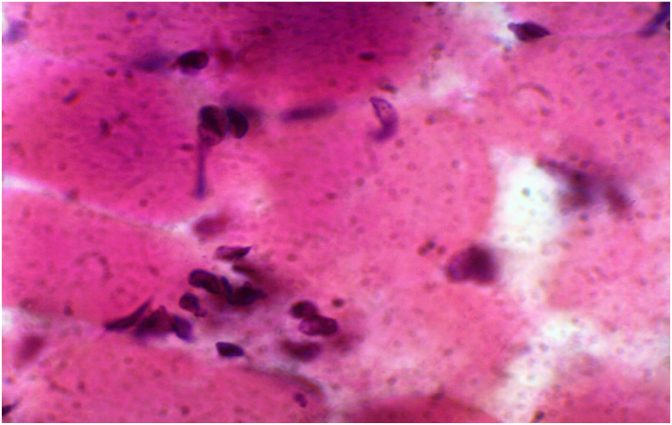
fibers are of equal size but there is an increased number of fibroblasts in between the muscle fibers.

ATPase at PH. 10.6, PH 4.6; PH. 4.2 showed an almost equal number of type I and type II fibers. The presence of prominent ring body vacuoles with Golgi type pattern in type II fibers was best seen at PH 10.6 (Fig. 3B).

**Fig. 3B:**
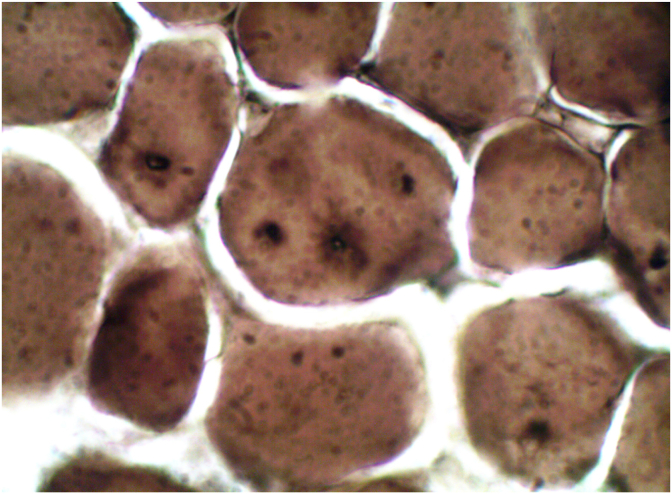
vacuoles in type II fibers with enhancing rim at ATPase 10.6.

NADH, Gomeri-trichome, Succinate dehydrogenase, Cytochrome oxidase, PAS all showed a normal staining pattern. The Dystrophy panel including Dystrophin, Dysferlin, Adhalin (alpha 1 sarcoglycan), Emerin showed a normal staining pattern.

## 4 Genetic study

An ETDA blood sample was taken and using next-generation sampling the results came out revealing a mutation in Chr8:100711831SNV, T > A in the VPS13B gene; p.leu2067 Ter.

The heterozygous mutation pattern seen in the VPS13B gene is a pathogenic variant that is usually seen in Cohen syndrome.

## 5 Discussion

Cohen syndrome is a very rare autosomal recessive syndrome that was first described in 1973, and the first VPS13B mutation was reported in 2003 [5]. Patients usually present with various clinical manifestations [6]. Few data and research have been conducted concerning Cohen syndrome and even though VPS13B mutation is considered an as underlying cause of Cohen syndrome, its diagnosis is still challenging due to the variation in the clinical presentations and phenotypic spectrum [10]. The usual method of testing or screening for Cohen syndrome is VPS13B next-generation sequencing. Clinical symptoms usually appear after the age of ten, therefore, it is a difficult disease to diagnose. We usually suspect Cohen syndrome in a patient if they have at least six of these features: retinal dystrophy, high myopia, microcephaly, developmental delay, joint hypermobility, typical Cohen syndrome facial gestalt, truncal obesity with slender extremities and neutropenia [10].

In this case, we have the first Jordanian case of Cohen syndrome to be reported; a novel heterozygous mutation in the VPS13B gene (p.leu2067 Ter) in one year and eight-month-old male twin patients born to first-degree consanguineous parents.

A muscle biopsy was taken for the first time as a diagnostic method. The muscle biopsy showed the increased and prominent Golgi pattern vacuolar ring in type II fibers in the muscle seen in all ATPase stains.

Patients with Cohen syndrome usually present developmental delay, intellectual disability, and eye manifestations with very diverse clinical findings on myopia retinal dystrophy [5].

Moreover, neutropenia is a common presentation of Cohen syndrome and it is associated with mild recurrent infections. Zhao et al. presented a cohort study where fifteen patients were studied and demonstrated mild to severe neutropenia in a constant fashion [7].

One of the most prominent symptoms our patients developed was salaam seizures. Salaam seizures also called infantile spasms are a rare type of epileptic seizure that affects infants with West syndrome [8]. They also presented with muscle dystonia and a decrease in their mental development. However, they have no eye manifestations although it being a common finding in patients with Cohen syndrome [9].

The VPS13B gene in Cohen syndrome (also known as COH1), is located on chromosome eight. It plays an essential role in multiple cellular functions that include preserving the integrity and function of the Golgi apparatus, protein glycosylations, and endosomal-lysosomal trafficking. [6]. In the case we presented, the patients were tested and diagnosed using next-generation sequencing which revealed a mutation in chromosome eight.

In this study, we talk about a rare case of twins with Cohen syndrome with a heterozygous mutation in the VPS13B gene. They presented with clear symptoms of Cohen syndrome and were the first Jordanian patients to be reported. A muscle biopsy was used as a diagnostic method. The limitations faced were the lack of studies and case reports concerning Cohen syndrome due to its rarity and diverse clinical symptoms for early diagnosis.

## 6 Conclusion

To the best of our knowledge, this is the first case report of Cohen syndrome from the Jordanian population. This report is unique, owing to the first muscle biopsy data available for a Cohen syndrome patient. The uniqueness of the presented case would make a good educational tool for the scientific community.

## Provenance and peer review

Not commissioned, externally peer-reviewed.

## Declaration of competing interest

No conflicts of interest.

## Registration of research studies

1. Name of the registry:
2. Unique Identifying number or registration ID:
3. Hyperlink to your specific registration (must be publicly accessible and will be checked):
